# Groundfish biodiversity change in northeastern Pacific waters under projected warming and deoxygenation

**DOI:** 10.1098/rstb.2022.0191

**Published:** 2023-07-17

**Authors:** Patrick L. Thompson, Jessica Nephin, Sarah C. Davies, Ashley E. Park, Devin A. Lyons, Christopher N. Rooper, M. Angelica Peña, James R. Christian, Karen L. Hunter, Emily Rubidge, Amber M. Holdsworth

**Affiliations:** ^1^ Institute of Ocean Sciences, Fisheries and Oceans Canada, Sidney, British Columbia, Canada V8L 5T5; ^2^ Department of Zoology, University of British Columbia, Vancouver, British Columbia, Canada V6T 1Z4; ^3^ Department of Forest and Conservation Sciences, University of British Columbia, Vancouver, British Columbia, Canada V6T 1Z4; ^4^ Pacific Biological Station, Fisheries and Oceans Canada, Nanaimo, British Columbia, Canada V9T 6N7; ^5^ Bedford Institute of Oceanography, Fisheries and Oceans Canada, Dartmouth, Nova Scotia, Canada B2Y 4A2

**Keywords:** climate change, regional ocean model, dissolved oxygen, hypoxia, depth, species distribution

## Abstract

In the coming decades, warming and deoxygenation of marine waters are anticipated to result in shifts in the distribution and abundance of fishes, with consequences for the diversity and composition of fish communities. Here, we combine fisheries-independent trawl survey data spanning the west coast of the USA and Canada with high-resolution regional ocean models to make projections of how 34 groundfish species will be impacted by changes in temperature and oxygen in British Columbia (BC) and Washington. In this region, species that are projected to decrease in occurrence are roughly balanced by those that are projected to increase, resulting in considerable compositional turnover. Many, but not all, species are projected to shift to deeper depths as conditions warm, but low oxygen will limit how deep they can go. Thus, biodiversity will likely decrease in the shallowest waters (less than 100 m), where warming will be greatest, increase at mid-depths (100–600 m) as shallow species shift deeper, and decrease at depths where oxygen is limited (greater than 600 m). These results highlight the critical importance of accounting for the joint role of temperature, oxygen and depth when projecting the impacts of climate change on marine biodiversity.

This article is part of the theme issue ‘Detecting and attributing the causes of biodiversity change: needs, gaps and solutions’.

## 1. Introduction

In marine ecosystems, climate change is expected to result in warmer waters and reduced dissolved oxygen, both of which are likely to impact organismal performance, population growth rates and viability [1,2]. There is evidence that marine species are more sensitive to temperature changes than terrestrial species, and many species are already shifting their distributions to track changing conditions [3–5]. Therefore, we expect to see considerable reorganization in the composition of marine communities as conditions continue to change [6,7]. Projections of how species and communities will respond to climate change over the coming decades are needed in order to develop management strategies for preserving biodiversity and ensuring the sustainability of fisheries and ecosystems [8].

In marine environments, species are strongly influenced by temperature and dissolved oxygen [1,2,9,10], both of which are projected to change in the coming decades (e.g. [11]). When available, experimental measurements of species-specific thermal tolerances and oxygen requirements, including how temperature influences oxygen requirements, can be used to assess how species distributions and abundances will shift as the oceanographic conditions change (i.e. via the metabolic index; [1,12,13]). However, because such measurements are not available for most marine fish, this approach cannot be applied to assess how climate change will impact the overall biodiversity and composition of marine communities (but see [14] for a potential alternative approach).

Instead, species distribution models (SDMs) can be used to leverage current species–environment associations across space to estimate species-specific temperature and oxygen responses [15]. SDMs are increasingly being applied in marine environments to project how species will respond to future conditions. These models generally project that species’ ranges will shift polewards as conditions warm [16]. Range shifts for species in west coast North American waters are projected to be particularly large, with many species’ range limits expected to change by more than 1000 km by the end of the century under high greenhouse gas emissions scenarios [17]. This result is consistent with estimates that suggest that the range edges of marine species are generally, but not universally, changing with warming ocean water temperatures [18,19]. In addition, there is evidence that some, but not all, marine species are shifting to greater depths as conditions warm [20]. In Canadian Pacific waters, there is evidence that changes in temperature and oxygen are impacting the distribution and densities of groundfish species [21], but that these changes have not yet resulted in major changes in the diversity and composition of the groundfish community [22].

The robustness of SDMs depends on properly accounting for, and distinguishing, the influence of multiple environmental variables on species distributions. Most SDMs to date do not account for oxygen (e.g. [17,20,23,24]), and in some cases rely on sea surface temperature when modelling species that live on the seafloor (e.g. [16,24]). In addition, demersal marine communities (e.g. groundfish) are highly depth-structured because of physiological limits to temperature, hydrostatic pressure [25] and hypoxia tolerance [9,26], as well as light, competition and food availability [27]. However, because depth, temperature and oxygen are closely correlated in many regions, it can be challenging to distinguish the role of each of these variables in determining contemporary species distributions. Some models have addressed this challenge by assuming that depth distributions are entirely determined by temperature, because this allows projected distributions to shift into deeper waters as conditions warm [17,28]. However, if depth—and associated variables (e.g. light and pressure)—influences species ranges directly, as well as by influencing water temperature and dissolved oxygen, then the direct effect of temperature and dissolved oxygen on species ranges cannot be determined without also accounting for the independent influence of depth.

Although climate SDM studies often focus on range shift rates and distances, climate change will also impact species within their range boundaries [21]. Projections that capture local-scale changes in species’ occurrences and abundances are particularly needed for the implementation of climate-resilient management strategies such as establishing networks of marine protected areas and fisheries management areas [8,29]. The recent development of regional ocean models that downscale climate projections at a high resolution (e.g. [11,30]) offers the opportunity to make projections at spatial resolutions (e.g. less than 3 km) that are relevant to marine spatial planning initiatives [31–33].

**Figure 1.**
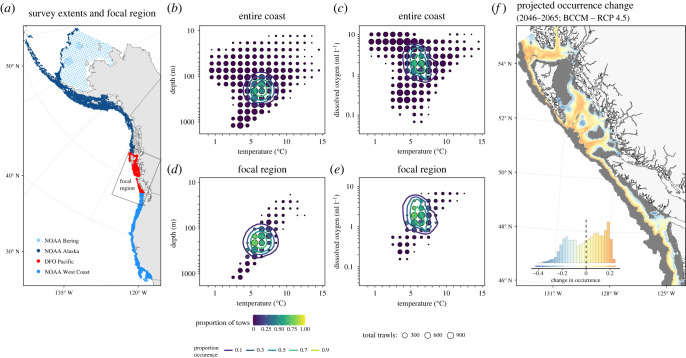
Overview of the SDM fitting and projection process using silvergrey rockfish (*Sebastes brevispinis*) as an example. (*a*) The extent of the groundfish survey data, with each point representing a single trawl, coloured by survey. Focal region outlined in (*a*) corresponds to the extent of the regional ocean models that are used for the future projections. The trawl data binned in environmental space for temperature by depth (*b*,*d*) and temperature by dissolved oxygen (*c*,*e*). The size of the circles shows the number of tows that fall into that bin, the colour shows the proportion of those tows where the species was present. The SDM model fit is shown as contour lines of predicted occurrence. (*b*,*c*) The full coastwide dataset was used to fit the SDMs. Panels (*d*) and (*e*) show only the trawls from BC and WA. (*f*) The projected change in occurrence for each 3 km^2^ grid cell between the historical baseline (1986–2005) and the future projection (2046–2065), here based on the BCCM model and RCP 4.5. The inset histogram in (*f*) shows the distribution of values across all 3 km^2^ grid cells and provides a legend for the colours shown on the map. Grid cells where the probability of occurrence was projected to be below 0.1 in both the historical and future time periods are shown in grey and are not included in the histogram, as we do not consider these to be important habitat for the species. Plots for all species and projected changes from both climate models and both RCP scenarios are available in electronic supplementary material, appendix A and in our Shiny app (https://msea.science/gf_climate_shiny/).

Here, we provide projections of groundfish community change in British Columbia (BC), Canada and Washington (WA), USA waters under future climate change scenarios (2046–2065) that account for the combined effects of temperature, dissolved oxygen, and seafloor depth at a spatial scale that is relevant to marine spatial planning and fisheries management. We do this by using SDMs to estimate how temperature, oxygen and seafloor depth determine the current occurrence and distribution of 34 groundfish species across the west coast from California to Alaska (figure 1*a*), based on fisheries-independent trawl surveys. We then combine these estimated species responses with two regional ocean models that cover BC and WA waters to project changes in species occurrences from a historical baseline of 1986–2005 to a future period, 2046–2065, under representative concentration pathways (RCP) 4.5 and 8.5 (figure 1). This time frame was selected because it is short enough to be relevant for informing policy decisions over the coming decades, while being long enough to ensure that changes in temperature and dissolved oxygen are unambiguously due to increased greenhouse gases. Estimating species’ responses to the environmental variables using the spatially extensive trawl survey dataset allows us to capture a much wider range of environmental conditions than are present in the region where we make our projections. In particular, the tight correlation between depth and temperature in BC and WA waters is not present across the coastwide dataset, and this allows us to separate the influence of these variables using correlational SDMs.

## 2. Methods

### (a) Estimating species response curves

#### (i) Fisheries-independent trawl data

We estimated how the observed distribution of groundfish species is determined by temperature, dissolved oxygen and seafloor depth using data from 31 239 fisheries-independent scientific research trawls. This dataset spans the entire American and Canadian west coast (figure 1*a*) and includes both species presence and absence in the trawls. The Canadian portion of the coast is surveyed by Fisheries and Oceans Canada (DFO) with the Groundfish Synoptic Bottom Trawl Surveys, which have been conducted since 2003 (DFO Pacific; 4218 trawls) [34,35]. The American portions of the coast are surveyed by the National Oceanic and Atmospheric Administration (NOAA) and include the NWFSC West Coast Groundfish Bottom Trawl Survey (NOAA West Coast; 10 769 trawls), which has been conducted since 2003, and the Alaska Groundfish Bottom Trawl Surveys in the Aleutian Islands, the Gulf of Alaska, the Bering Sea upper continental slope, and the east and north Bering Sea shelf [36,37]. The Alaska Groundfish Bottom Trawl Surveys have been conducted since the 1980s, but our analysis only includes data from 2000 onward in order to align with the temporal extent of the DFO and NOAA West Coast surveys. Because surveys in the sub-regions of Alaska use different protocols, our analysis considers them as two separate surveys: the surveys on the Bering Sea shelf (NOAA Bering; 7365 trawls) and those in the Aleutian Islands, the Gulf of Alaska and the Bering Sea upper continental slope (NOAA Alaska; 8887 trawls). We included data from all surveys up to 2019. We included all 77 groundfish species that were present in the overall trawl dataset that did not show obvious breakpoints in occurrence that corresponded with the boundaries of the three surveys. However, our final analysis included only the 34 species (listed in the relevant figures) for which the models had adequate forecasting ability, as outlined in the ‘Assessing predictive accuracy’ section 2a(iii) below.

Temperature, depth and dissolved oxygen for the observed data were obtained by CTD instrumentation and dissolved oxygen sensors deployed on the headrope of the trawls. Dissolved oxygen was available for 17.7% of the trawls. We filled in missing oxygen data using predictions from a generalized linear mixed effects model fitted with the sdmTMB package [38] which was also informed by an additional 7037 oxygen observations from the International Pacific Halibut Commission (IPHC) ocean profile data [39]. Full details of the dissolved oxygen model are provided in the electronic supplementary material. This model was able to predict withheld data with an *R*^2^ of 0.952 (electronic supplementary material, figures S1 and S2). The correlation between log depth and temperature was −0.904 within our focal region but was 3 × 10^−4^ across the full dataset. The correlation between log depth and log dissolved oxygen was −0.811 within our focal region and was −0.864 across the full dataset. The correlation between temperature and log dissolved oxygen was 0.742 within our focal region and was −0.221 across the full dataset.

#### (ii) Species distribution model

For each species, we modelled occurrences in the coastwide trawl dataset using a generalized linear model with a binomial distribution and a logit link function to map the linear predictors to the binary presence/absence data using the sdmTMB package [38] in R v. 4.0.2 [40]. The fixed effects were temperature, log dissolved oxygen, log depth and survey. We included quadratic terms for temperature and log depth to allow species occurrences to peak at intermediate values (figure 1*b*–*e*). We fitted a breakpoint function for log dissolved oxygen to reflect the fact oxygen is a limiting factor [26]. That is, each species is expected to have an oxygen threshold, below which oxygen limitation is expected to reduce their probability of occurrence, but above this threshold species should not be sensitive to changes in oxygen concentrations [41]. A survey term specifying the data source—NOAA West Coast, NOAA Alaska, NOAA Bering or DFO Pacific—was included as a catchability covariate to account for variation in detection probability across surveys due to differences in survey design and gear. We expected that these differences in detection probability should be small because our analysis was based on the presence/absence data. The use of survey ID as a fixed effect in a presence/absence model has been demonstrated to sufficiently capture catchability differences between trawl and longline surveys [42], where differences in catchability are expected to be considerably larger than when comparing across surveys that all use similar trawling methods. Although additional variables such as substrate and rugosity are known to be important determinants of groundfish distribution [22,43], we elected not to include them because we lack data for these variables that span the entire spatial distribution of the groundfish dataset. We elected not to include spatial or spatio-temporal random fields in our models. While random fields offer an effective way of accounting for unmeasured environmental variables [38], they have the potential to absorb variation in species occurrences that is due to the environmental covariates, and thus result in an underestimate of species’ sensitivity to environmental change [44–46].

Although model comparison using a range of SDM types (e.g. MAXENT, random forest, GAMS) is recommended when making predictions for contemporary distributions [47], we elected not to take this approach because it risks selecting a model that is overfitted or includes unreasonable model parameters for our fixed effects. Instead, we opted to construct models based on ecological principles and knowledge of the variables that are likely to be important for determining species’ responses to climate change.

In the majority of species, our models estimated reasonable dissolved oxygen responses—that is, positive oxygen slopes, a breakpoint that fell within the range of observed oxygen conditions (i.e. less than 10 ml l^−1^), and proper model convergence. However, for some of the species, the breakpoint model estimated a negative slope above the breakpoint. We assume that this was not a real response to increasing oxygen but was due to the fact that depth and oxygen are correlated and so the model is erroneously attributing the fact that the species is not found in shallower waters (where oxygen tends to be higher) to oxygen. For species that did not meet this criteria, we elected to drop the oxygen response and model their occurrence based on temperature, depth and survey.

#### (iii) Assessing forecasting accuracy

We assessed the forecasting accuracy of the SDM by comparing how well a model fitted to only data from 2000 to 2010 could forecast species’ occurrences in trawls within our focal region for the period of 2011–2019. This assessment approximates the approach that we used in our projections (described below), but uses the latter half of our trawl data as testing data in order to estimate how well our models can predict future time periods that were not included in the training data. In particular, this post-2010 period includes the marine heatwave that occurred in the region from late 2013 to 2016 [48], and so provides a test of how well the model can predict anomalous conditions without historical precedent. The final set of species included in this manuscript includes only those species that exceeded a threshold Tjur *R*^2^ [49] of 0.2 and an area under the curve (AUC; [50]) of 0.75 based on the temporal forecasting accuracy assessment. Tjur *R*^2^ quantifies the mean difference in forecasted occurrence between trawls where the focal species is present and trawls where it is absent [49] and so assesses how good the model is at distinguishing presence/absence patterns. Note that this temporal blocking of training and testing data was only used for assessing the predictive accuracy of the models; the models used to fit the SDMs that were used to make the projections for the 2046–2065 period (described in subsequent sections) included all trawls in the dataset.

### (b) Projecting groundfish biodiversity changes in British Columbia and Washington waters

#### (i) Regional ocean models

We based our groundfish biodiversity change projections on two regional models that downscale climate projections: the British Columbia Continental Margin model (BCCM; [30]) and the North-Eastern Pacific Canadian Ocean Ecosystem model (NEP36-CanOE; [11]). The BCCM model is an implementation of the Regional Ocean Modeling System (ROMS; [51]) at a 3 km horizontal resolution. The NEP36 model is an implementation of Nucleus for European Modelling of the Ocean (NEMO) numerical framework 3.6 [52] at a variable grid spacing between 1.5 and 2.25 km. Both models used anomalies from the Canadian Earth System Model v. 2 [53] at the open boundaries and the Canadian Regional Climate Model v. 4 (CanRCM4; [54]) for the surface boundary conditions to downscale climate projections.

The BCCM model outputs were interpolated from a curvilinear to a regular 3 km grid using a thin plate spline using the fields [55] and raster packages [56]. The NEP36 model outputs were interpolated to the same 3 km grid using a linear interpolation. We used a historical baseline of 1986–2005 because these years were present in the historical hindcast of the BCCM as well as the historical climatology of the NEP36. We used projected values from these models for 2046–2065 based on RCP 4.5 and 8.5. RCP 4.5 represents a scenario with moderate climate change mitigation and RCP 8.5 represents a no mitigation, worst-case scenario [53,57,58]. We elected to use 20-year climatologies for the historical baseline and future scenarios to reduce the influence of year-to-year variation in climate (i.e. natural variability).

We used mean summer (April–September) near-bottom temperature and dissolved oxygen averaged across all years in the historical baseline and future projection periods (electronic supplementary material, figures S3–S5). Historical temperatures from both models and dissolved oxygen from the BCCM model were comparable to those observed in the research trawl surveys, but oxygen concentrations from the NEP36 model were consistently high. Therefore, we bias-corrected the NEP36 data based on the BCCM hindcast as a common baseline. We elected to use proportional change rather than absolute change for this bias correction to avoid negative projected oxygen concentrations. We did this by calculating the proportional change in oxygen and temperature between the historical and future projections for each 3 km^2^ grid cell as: *x*_f_/*x*_h_, where *x*_f_ represents the future projected value and *x*_h_ represents the historical value [59]. We then multiplied these proportional changes by the historical BCCM values to obtain future projections that were bias-corrected to the BCCM baseline. We calculated proportional changes in temperature in kelvin and then converted these back to celsius to avoid issues of estimating proportional increases for values close to zero.

#### (ii) Species-level projections

Using the models that we validated in our forecasting accuracy assessment, we projected the occurrence of each species in each 3 km^2^ grid cell for the historical baseline, as well as for two emissions scenarios, from each of the two regional ocean models. We substituted the *in situ* temperature, oxygen and depth measurements from the trawl surveys with outputs from the regional oceanographic models. This substitution requires making the assumption that the *in situ* measurements are comparable with the model outputs. The model outputs represent mean summer (April–September) conditions over multiple years and so are less variable than the *in situ* measurements. However, a comparison of outputs from the BCCM model and the *in situ* measurements shows good agreement, with a correlation of 0.841 for temperature and 0.836 for dissolved oxygen (electronic supplementary material, figure S6). A similar comparison is not possible for the NEP36 model, because we do not have model outputs from after 2005. For our projections, we set our survey fixed effect to be DFO Pacific as this survey covers the majority of our focal region.

Projected occurrence change for each scenario and model was calculated as the historical projected occurrence subtracted from the future projected occurrence for each grid cell (figure 1*f*). For each species, we excluded grid cells where the projected probability of occurrences fall below 0.1 in both the historical and future periods, as these are not considered to be important habitat. This allowed us to exclude areas where the projected change in occurrence is minimal because conditions are unsuitable in both time periods. Our findings remain qualitatively unchanged when we use a threshold of 0.3 (results not shown).

To estimate the degree to which each species could tolerate increases in temperature, decreases in oxygen and shifts to deeper depths, we estimated a margin for change across all of the trawls in our focal region for which the species was present. This allowed us to assess which aspects of environmental change are most likely to have negative consequences for each species, within our focal region. This metric is akin to a thermal-safety margin [60,61], but relies on modelled, rather than physiologically based, tolerance limits, and it can be applied to other niche axes such as dissolved oxygen and depth. This margin for change was estimated as the difference between the conditions in which they were observed (i.e. present in a trawl) in BC and WA and a given threshold for each variable. This threshold was the estimated breakpoint for dissolved oxygen and the maximum temperature or depth where our SDM estimates that the probability of occurrence remains above 0.1. However, because the influence of one variable on occurrence probabilities depends also on the other two variables, we estimated these thresholds for temperature and depth based only on combinations of conditions observed in our coastwide training dataset. This ensured that thresholds were not based on unreasonable combinations of variables (e.g. high dissolved oxygen or temperature at depth). We also excluded conditions that were greater than 50 m of the maximum depth and greater than 1°C of the maximum temperature where the focal species was observed to be present in the coastwide dataset. Once we had estimated the threshold values for each variable, we compared these with the observed conditions in all of the trawls in our focal region (i.e. BC and WA), where the focal species was observed. The difference between the observed conditions, conditional on species presence, and the estimated threshold was then taken as the margin for change in that location and time. We then aggregated the margin for change across all trawls to obtain a distribution for each species, for each environmental variable, for our focal region.

We quantified projection uncertainty due to SDM uncertainty as the average width of the 95% confidence interval for the probability of occurrence. For this, we took the average value across all grid cells where the species was projected to be present with at least a probability of 0.1 in the future scenarios, averaged across both regional ocean models and both emissions scenarios. We quantified regional ocean model uncertainty as the absolute difference in projected occurrence in the future scenarios based on the two regional ocean models, averaged across all grid cells where the species were projected to be present with at least a probability of 0.1, and averaged across both emissions scenarios. We quantified scenario uncertainty as the absolute difference in projected occurrence between RCP 4.5 and 8.5, averaged across all grid cells where the species was projected to be present with at least a probability of 0.1, and averaged across both regional ocean models.

#### (iii) Community-level projections

Projected species richness for each period, regional ocean model and scenario was calculated as the summed probability of occurrences across all species. Projected species richness change was then calculated as the historical projected richness subtracted from the future projected richness within each 3 km^2^ grid cell.

## 3. Results

### (a) Forecast accuracy

**Figure 2.**
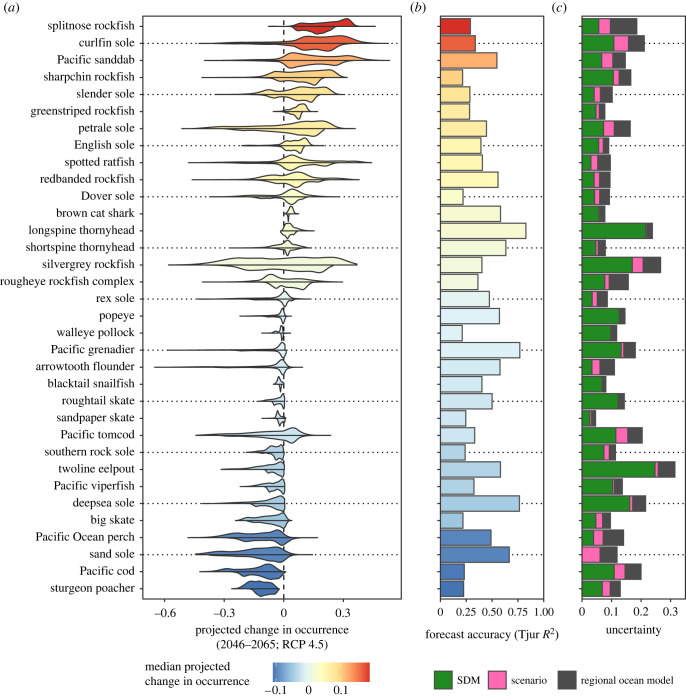
Distributions of projected change in occurrences across the 3 km^2^ grid cells in the study region for all species (*a*) based on a comparison of average conditions in 1986–2005 versus 2046–2065 under the RCP 4.5 emissions scenario (see electronic supplementary material, figure S10 for RCP 8.5). For each species, the upper half of the distribution shows projections based on the NEP36 model and the lower half shows projections based on the BCCM model. Grid cells where projected occurrences fall below 0.1 in both the historical and future periods are not considered to be habitat, and changes in occurrence in these grid cells are excluded from the distributions. Species are ordered from most positive to most negative median change in projected occurrence. (*b*) The forecast accuracy, measured as Tjur *R*^2^ for post-2010 data in the focal region using a model trained on all pre-2011 data. The colour indicates the median projected change in occurrence across all 3 km^2^ grid cells in the region based on the BCCM RCP 4.5 scenario. (*c*) The average estimated uncertainty of the projections across all 3 km^2^ grid cells where the probability of occurrence is greater than 0.1 in both the historical and future periods, separated by SDM uncertainty (95% confidence interval), emmissions scenario difference and regional ocean model difference.

Of the 77 species that we assessed for forecast accuracy, 34 had a Tjur *R*^2^ greater than our 0.2 threshold for inclusion (electronic supplementary material, figure S7). All species that met this threshold also had an AUC greater than 0.75. The mean and maximum Tjur *R*^2^ of the species that met our inclusion threshold were 0.43 and 0.83, respectively (figure 2*b*). Models that included a subset of the environmental covariates generally had lower forecast accuracy (electronic supplementary material, figure S8), with the greatest loss of predictive accuracy occurring with the omission of depth, survey and temperature. Models that did not include dissolved oxygen had similar forecast accuracy compared with models with dissolved oxygen (electronic supplementary material, figure S8). However, we opted to keep oxygen in our models because experimental evidence clearly shows that oxygen is an important determinant of species performance [1].

### (b) Estimated environmental responses

Occurrence probabilities for all species were estimated to peak at intermediate depths (i.e. negative quadratic depth coefficient), but there was wide variation in the estimated depth ranges (i.e. variation in linear and quadratic depth coefficients; electronic supplementary material, figure S9). Our models estimated reasonable oxygen breakpoints and slopes for 22 out of the 34 species. The estimated oxygen breakpoints varied from 0.154 to 2.366 ml l^−1^, with a median of 0.802 ml l^−1^ (electronic supplementary material, figure S9). Occurrence probabilities for all but four species were estimated to peak at intermediate temperatures (i.e. negative quadratic temperature coefficient), but there was wide variation in the estimated temperature ranges (i.e. variation in linear and quadratic temperature coefficients; electronic supplementary material, figure S9). The four species that were not estimated to have unimodal temperature responses were all species where occurrences were highest at the lowest temperatures (i.e. lowest linear temperature coefficients).

### (c) Projected environmental change

Both regional ocean models projected increases in bottom temperature across the entire focal region, except for off the shelf at depths greater than 1400 m, where increases are projected to be less than 0.01°C (electronic supplementary material, figure S4). The NEP36 model projects a greater degree of warming (median 0.98°C for RCP 4.5) compared with the BCCM (median 0.73°C for RCP 4.5). Projected changes are greater under RCP 8.5 (median 1.21°C for NEP36 and 0.92°C for BCCM). The greatest increases (as high as 2.82°C under RCP 8.5 in the NEP36 model) are projected in the shallowest waters to the east of Haida Gwaii.

Projected changes in dissolved oxygen differed more between the two regional models (electronic supplementary material, figure S5). The BCCM model projected oxygen losses in the shallower waters on the continental shelf (less than 300 m; median −0.12 ml l^−1^ under RCP 4.5 and −0.16 ml l^−1^ under RCP 8.5), negligible changes in oxygen at mid-depths (troughs in Queen Charlotte Sound and on the shelf slope), and increases at the deepest waters off the shelf (greater than 500 m; median 0.03 ml l^−1^ under RCP 4.5 and 0.03 ml l^−1^ under RCP 8.5). By contrast, the NEP36 model projected decreases across most of the region (median −0.25 ml l^−1^ under RCP 4.5 and −0.27 ml l^−1^ under RCP 8.5; maximum −0.63 ml l^−1^ under RCP 4.5 and −0.72 ml l^−1^ under RCP 8.5).

### (d) Projected species’ occurrence change

Projected occurrence changes for BC and WA varied considerably across species (figure 2*a*). These projections were consistent across the two regional ocean models, although the projected changes based on the NEP36 model tended to be slightly larger compared with those based on the BCCM model (figure 2*a*). Likewise, the projected changes in species occurrences were similar in models based on the RCP 4.5 and 8.5 emissions scenarios, with RCP 8.5 resulting in projected changes that were of slightly greater magnitude (figure 2 versus electronic supplementary material, figure S10). Under the RCP 4.5 scenario with the BCCM (NEP36) model, 5 (8) species were projected to decrease in occurrence in at least 95% of the region where they were estimated to be present, 3 (3) species were projected to increase in occurrence in at least 95% of the region where they were estimated to be present, while the remaining 26 (23) species were projected to increase and decrease in prevalence in different parts of the region. The projections were similar for the RCP 8.5 scenario, with 5 (10) species decreasing in occurrence, 3 (3) species increasing in occurrence and 26 (22) showing variable changes in projected occurrence.

Quantifiable uncertainty in the probability of occurrence change varied across species, ranging from 0.046 for sandpaper skate to 0.545 for sand sole, with an average of 0.154 across all species (figure 2*c*). Uncertainty in projected occurrences from SDM uncertainty resulted in the greatest variation in projected occurrences, with an average 95% confidence interval width across all grid cells (with a projected occurrence >0.1) and species of 0.096. The next greatest source of uncertainty was variation in projected occurrences across the two regional ocean models, which had an average difference of 0.038 across all grid cells (with a projected occurrence >0.1) and species. Emission scenario differences contributed the least uncertainty to our projections, with an averaged difference of 0.021 across all grid cells (with a projected occurrence >0.1) and species.

### (e) Species’ margin for change

**Figure 3.**
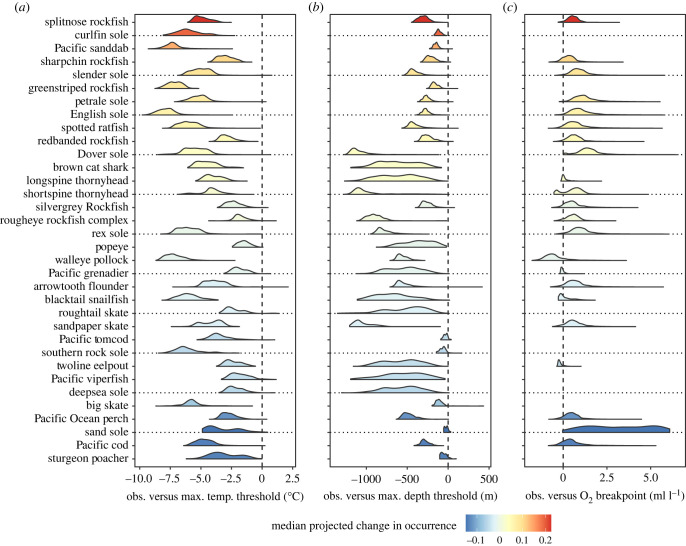
Species’ margin for change in temperature (*a*), depth (*b*) and dissolved oxygen (*c*). Margins were calculated as the observed environmental value in each trawl where a species was present minus the threshold (breakpoint for dissolved oxygen) value for that variable estimated using the SDM (see Methods for details). Threshold values are for high temperature and depth and for low dissolved oxygen concentrations. Distributions represent the margins for all trawls where a species was present. The distance between the distribution and the dashed 0 line (left for temperature and depth, right for oxygen) indicates the degree to which the species is estimated to be able to tolerate warmer, deeper, or lower oxygen conditions, respectively. Species that do not have distributions for oxygen margin change are those that did not have oxygen included in the model. As for figure 2, the colour indicates the median projected change in occurrence between 1986–2005 and 2046–2065 for the RCP 4.5 scenario in the BCCM model across all 3 km^2^ grid cells in the region. Species are ordered from most positive to most negative change.

There was considerable variation in how far species in BC and WA waters are from our estimated maximum temperature thresholds (figure 3*a*). Species such as greenstriped rockfish and English sole were caught (more than 99% of trawls) in conditions that are at least 5°C cooler than their estimated maximum temperature threshold. Others, such as Pacific viperfish, roughtail skate and sand sole, were found at temperatures right up to, and sometimes exceeding their maximum temperature threshold. By contrast, groundfish in BC and WA tended to extend all the way to their estimated maximum depth thresholds (figure 3*b*; walleye pollock and rex sole being notable exceptions). Observed species’ occurrences were common above the estimated oxygen breakpoints, but tended to decrease quickly at lower oxygen levels (figure 3*c*). However, some species, such as walleye pollock, blacktail snailfish and twoline eelpout, were predominantly found in conditions where oxygen was estimated to be limiting. In general, the species that were projected to increase in occurrence throughout BC and WA are those that were far from their temperature thresholds (figure 3*a*). Species that were projected to have mixed responses under future conditions are those that have wide depth ranges (figure 3*b*). Species that were projected to decrease in occurrence throughout the region fall into two categories: (1) they are close to their maximum temperature thresholds and occupy a relatively narrow range of depths (e.g. sand sole, sturgeon poacher), (2) they are already found in conditions with oxygen concentrations near or below their estimated oxygen breakpoint (e.g. Pacific cod, Pacific Ocean perch).

#### (i) Projected species richness change

**Figure 4.**
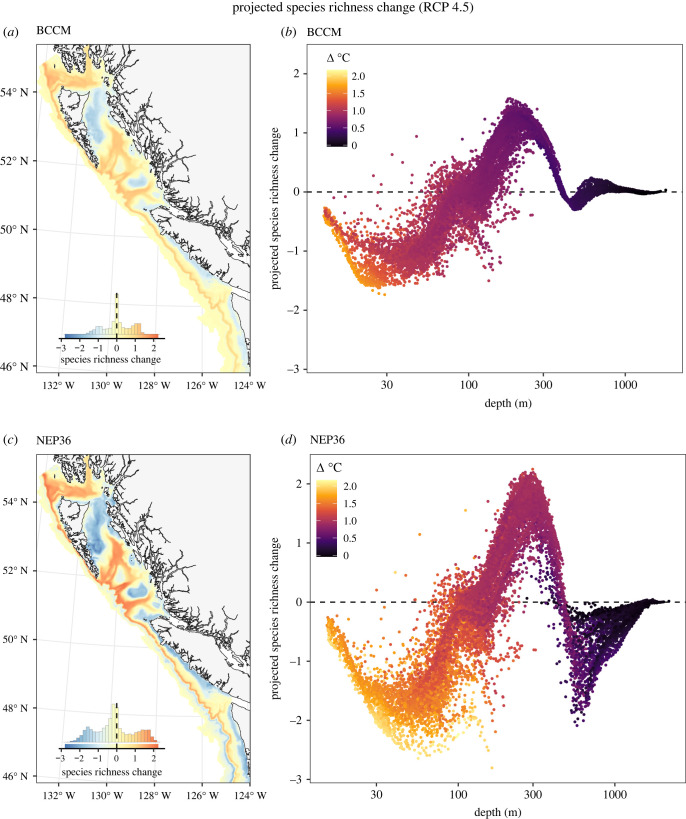
Projected species richness change (*a*,*c*) mapped across the study region between historical (1986–2005) and future (2046–2065, RCP 4.5) conditions and as a function of seafloor depth (*b*,*d*). (*a*,*b*) Projections based on the BCCM model. (*c*,*d*) Projections based on the NEP36 model. The inset histograms in (*a*,*c*) show the distribution of values across all 3 km^2^ grid cells and provide a legend for the colours shown on the maps. Each point in (*b*,*d*) represents a single 3 km^2^ grid cell in (*a*,*c*), coloured by the projected temperature change. See electronic supplementary material, figure S11 for results based on RCP 8.5.

Projected species richness changes varied spatially across our focal region, ranging from −4.2 to 2.6, with a median of 0 across both regional ocean models and both emissions scenarios (figure 4*a*,*c*; electronic supplementary material, figure S11a,c). These projected richness changes varied strongly with depth (figure 4*b*,*d*; electronic supplementary material, figure S11b,d). Decreases were projected in the shallowest waters (less than *ca* 100 m), where warming is projected to be greatest, and in deep, low-oxygen waters (deeper than *ca* 600 m), where further reductions in oxygen are projected to be detrimental for many species. By contrast, species richness is projected to increase at mid-depths (*ca* 100–600 m) as species shift to greater depths to deal with warming or shift to shallower depths to avoid hypoxic conditions. Projected changes were greater in magnitude based on the NEP36 model, particularly at depth, where the NEP36 model projects oxygen loss while the BCCM projects oxygen increases over this time period (electronic supplementary material, figure S5). Projected richness changes were similar, but of greater magnitude, for the RCP 8.5 emissions scenarios (electronic supplementary material, figure S11).

## 4. Discussion

Our analysis suggests that projected warming and deoxygenation is likely to cause a reorganization of the groundfish community in BC and WA by 2046–2065. We estimate that species will differ in their responses to climate change; some species will benefit from warmer conditions and increase in occurrence, others will shift to deeper waters as conditions warm, but those that cannot tolerate greater depths or lower oxygen are expected to decrease in occurrence. While species that are projected to decline are roughly balanced by species that are projected to increase, the overall impact on the community is expected to depend on depth. At depths of less than 100 m, where warming is projected to be greatest, we expect to see a decline in species richness. Species richness is also projected to decline at depths below 600 m because of oxygen loss. However, there is uncertainty in how much richness will decline owing to oxygen loss as our two regional ocean models differ in their projections of oxygen change at these depths. At mid-depths, between 100 and 600 m, we expect species richness to increase as warming drives shallow species deeper and deoxygenation in deeper waters pushes species shallower. The increase in species richness is projected to be greatest at depths around 300 m as below this hypoxic conditions [62] may prevent further species range shifts. While the magnitude of these projected changes to the groundfish community varies depending on the regional ocean model and the emissions scenario used, the overall pattern of change is generally consistent in all cases. This result suggests that our projections for how the groundfish community will reorganize should be qualitatively robust to uncertainty in how climate change will play out over the coming decades.

These findings demonstrate the importance of accounting for the influence of depth and dissolved oxygen when projecting how climate change will affect species in marine environments. Based on our projections, it is not possible to determine how a species will respond to warming based only on its temperature response curve because species vary in the degree to which their tolerance to depth and low oxygen constrains their ability to shift to greater depths as conditions warm (figure 3). Therefore, SDMs that do not account for depth and dissolved oxygen (e.g. [17]) may underestimate the sensitivity of marine organisms to climate change because they assume that species can always shift to deeper waters when conditions warm. By contrast, models that rely only on sea surface temperatures (e.g. [16]) assume that species cannot shift in depth to deal with warming; thus they are likely to overestimate the sensitivity of groundfish to climate change. The fact that dissolved oxygen and depth were correlated, even in our coastwide dataset, posed a challenge for accurately estimating the oxygen responses in our models. For a subset of species, our models estimated that occurrence decreased with increasing oxygen, which is unlikely given the fact that oxygen is understood to be a limiting factor [41]. This indicates that, for these species, the models are erroneously attributing the fact that the species is not found in shallower oxygenated waters to oxygen rather than some other variable that is correlated with depth. Indeed, models without oxygen responses tended to have similar forecast accuracy to those with oxygen responses, indicating that the depth response is able to capture the oxygen response, at least over the time period for which we have trawl data. However, these simpler models that do not include oxygen, despite having similar ability to forecast contemporary species distributions, unrealistically assume that species will not respond to future changes in oxygen. Therefore, because oxygen limitation is understood to have strong and detrimental impacts on fish performance [1,41], and including oxygen does not reduce the ability to forecast contemporary distributions, we elected to keep oxygen in our SDMs whenever it was possible to estimate reasonable parameters for the oxygen response.

Of course, additional variables that we have not accounted for may further constrain species distributions as well as their responses to warming and deoxygenation. For example, projected ocean acidification [11,30] may impact fish populations [63,64], but SDMs are not well suited to projecting these because we lack occurrence data across relevant pH ranges. Species distributions may also be constrained to certain seafloor substrate types [65] and including substrate in our models may result in more accurate projections (e.g. [17]). However, the lack of comprehensive substrate data for the entire extent of the groundfish surveys used in our analysis precluded us from including this variable in our models. It is likely that the inclusion of additional variables would have improved model performance, and may have increased the number of species that met our inclusion criteria based on the temporal forecasting assessment. However, many of the unobserved variables also change with depth so, as implemented here, depth may be a proxy for these processes. A previous study of groundfish in BC estimated that the addition of oceanographic variables, including substrate, currents, and primary production, to a model that already included depth only resulted in a minor increase in the accuracy of predictions [22].

By combining spatially extensive scientific survey data with regional ocean models, we were able to develop robust models that are informed by broad spatial and environmental gradients but make projections at a relatively high resolution. If we had trained our SDMs on data only from our focal region we would not be including the full range of environmental conditions that define the distributions of these species. Furthermore, the high correlation between depth, temperature and oxygen in BC and WA would have prevented us from distinguishing their unique influence on species’ distributions [22]. While some species in our models have ranges that extend beyond the broad spatial extent and environmental range sampled by the surveys, we expect that this had negligible impact on our projections because our focal region is situated in the middle of the geographical and environmental space sampled by the surveys. Reassuringly, the future environmental conditions that are projected by our regional models fall within the range of conditions present in our survey data, except in the few warmest grid cells, where future conditions are projected to exceed our observational range by less than 1°C (electronic supplementary material, figure S12). Thus, we are largely avoiding extrapolating into novel climate conditions, which is a key source of uncertainty in many SDM projections [66]. By contrast, if we had extended our projections to cover the full spatial extent of the survey data, we would have had to use low-resolution outputs from global climate models, which have not been downscaled to incorporate the complex bathymetry of the continental shelf [67,68]. Alternatively, we could have stitched together multiple regional ocean models (e.g. [69–71]), but this would have required additional assumptions because models differ in their underlying mechanics, data and assumptions. As our goal was to make projections that could be used for ongoing management initiatives in Canadian Pacific waters, this approach was not necessary.

Projecting biodiversity responses to climate change involves considerable uncertainty [72] and our approach allows us to quantify some aspects of this. Of the uncertainty that we could quantify, roughly half was due to uncertainty in our SDMs and the remainder was due to regional ocean model uncertainty or scenario uncertainty. This amount of uncertainty in the SDMs is typical [73], stemming from the fact that contemporary species distributions are also influenced by other factors that we have not included in our model. In addition, although oxygen demand is understood to vary with temperature [1], limitations in the implementation of breakpoint models prevented us from estimating a temperature-dependent oxygen breakpoint. However, although somewhat unrealistic, this limitation is unlikely to have greatly increased the uncertainty in our SDMs because low oxygen concentrations occurred almost exclusively at depths where temperature variation and projected change was small.

To reduce uncertainty due to year-to-year variation in climate, our model projections are based on 20-year climatologies with a future period that is far enough ahead to ensure that changes are unambiguously due to greenhouse gases [11,74]). We have made projections based on two different emissions scenarios, and two different regional ocean models that are both downscaled from the same global model, the second generation Canadian Earth System Model (CanESM2), using different downscaling techniques. While the BCCM model was run inter-annually and then averaged to produce the climatologies [30], the NEP36 model used atmospheric climatologies with augmented winds to force the ocean model and produce representative climatologies [11]. Comparing these regional projections provides an estimate of the uncertainty across different regional downscaling models and methods. We find that the projected impacts of climate change on the groundfish community are more sensitive to the differences in the regional ocean models than they are to the emissions scenarios used. However, these differences are in magnitude (changes tend to be larger based on NEP36 compared with the BCCM) rather than in direction, with both models resulting in similar overall patterns of biodiversity change and turnover for the groundfish community. Over the 60-year time period (1986–2005 versus 2046–2065) used in our study, our projections suggest that groundfish community changes are similar regardless of the scenario used. This is not surprising given the fact that the two emissions scenarios do not appreciably differ until later in the 21st century [53]. Ideally, we would also quantify uncertainty due to differences between global climate models and use the ensembles from each of the models (e.g. [17,75]); however, regional models driven by other global models have not yet been developed for this area. We expect that projections based on different global climate models would show greater variation than we see between our regional ocean models, but they would produce similar overall patterns in projected groundfish community change unless they project vastly different temperature and oxygen changes.

One aspect of projection uncertainty that we cannot quantify is that which is due to eco-evolutionary processes [72]. Interactions between species, movement across the seafloor (e.g. dispersal, migration, foraging [76]), and evolution all influence current species distributions as well as the way that species will respond when conditions change [77–79]). However, SDMs implicitly, and unrealistically, assume that the influence of interactions between species will not change as species shift their distributions and respond to future environmental conditions, that species are not dispersal-limited, and that they will not adapt or acclimatize to environmental conditions. Our projections suggest that warming and deoxygenation will cause species to shift their depth ranges, causing more overlap in ranges at mid-depths. How this increase in species richness at these depths (and the reductions of species richness in shallower and deeper waters) alters food web dynamics and is a major source of uncertainty [6,80]. For example, our projections assume that prey exist in sufficient abundance at those new depths, and that the species will be able to establish in the face of novel competitors and predators [81]. Likewise, any degree of acclimation or adaption to environmental change would cause us to overestimate changes in areas where occurrences are projected to decrease [82]. Despite these unavoidable sources of uncertainty, we can use our understanding of eco-evolutionary community dynamics to identify areas where our projections are likely to have more and less uncertainty. That is, eco-evolutionary dynamics lead to the greatest uncertainty at range edges, where they influence whether an established species will be lost, as well as whether a new species can establish [25,72,83]. Conversely, eco-evolutionary processes should result in less uncertainty in areas that are projected to remain as suitable habitat for a species. These areas that are projected to remain suitable offer a ‘no regrets’ conservation opportunity as their protection is likely to benefit the species, both now and in the future.

The species in our analysis are all under some degree of fishing pressure—either as the target of fisheries or as bycatch [34]. Climate change will make the management of these species more challenging and is likely to threaten the sustainability of some fisheries [84]. Estimated species’ sensitivity to climate change is a key knowledge gap in current groundfish stock assessments in this region. While changes in biomass are likely to differ from the changes in occurrence that we have projected here [22], we expect occurrence changes to be informative of the sensitivity of species to climate change. Thus, our results can be used to integrate climate change risk into stock assessments and ecosystem-based fisheries management in this region [84,85]. For many species, this may require accounting for climate-driven shifts in species distributions and biomass across international boundaries [86].

With this analysis, we have demonstrated how spatially extensive scientific survey data can be combined with high-resolution regional ocean models to provide projections of groundfish community changes at a scale that is relevant to developing spatial management strategies to preserve marine biodiversity and sustainable fisheries. Furthermore, we have demonstrated that our overall projected patterns of biodiversity change are consistent regardless of the level of emissions, and across the two different regional ocean models that are available for our region. However, had we chosen a more distant future time period, the sensitivity to emissions scenario would have been greater. These projections can inform ongoing management initiatives to ensure that they account for anticipated impacts of climate change. For example, Canada is in the process of establishing a network of marine protected areas (MPAs) and refuges to provide long-term conservation of biodiversity [87]. While climate change adaptation principles are largely lacking from Canadian MPA management plans to date [29], our projections offer an opportunity to ensure that new protected areas in BC are situated in areas that will benefit groundfish and other species, both now and under future climates.

## Data Availability

Groundfish Survey Data from the DFO Groundfish Synoptic Bottom Trawl Surveys are available at: https://open.canada.ca/data/en/dataset/a278d1af-d567-4964-a109-ae1e84cbd24a. Data from the NOAA U.S. West Coast Groundfish Bottom Trawl Survey are available at: https://www.webapps.nwfsc.noaa.gov/data/map. Data from the NOAA Alaska Groundfish Bottom Trawl Surveys are available at: https://www.fisheries.noaa.gov/alaska/commercial-fishing/alaska-groundfish-bottom-trawl-survey-data. Regional Ocean Model Data monthly averages for the NEP36 model are available at: https://open.canada.ca/data/en/dataset/a203a06d-9c1f-4bb1-a908-fc52912ff658. Historical climatologies from the BCCM are available at: https://open.canada.ca/data/en/dataset/1084a522-c2dd-47a0-81ac-d4810ce29056. All code used for this analysis is available at: https://gitlab.com/dfo-msea/cc_sensitivity_predict.
